# Maternal age and maternal environment affect egg composition, yolk testosterone, offspring growth and behaviour in laying hens

**DOI:** 10.1038/s41598-022-05491-6

**Published:** 2022-02-03

**Authors:** Tina M. Widowski, Leanne Cooley, Simone Hendriksen, Mariana Roedel Lopez Vieira Peixoto

**Affiliations:** 1 Department of Animal Biosciences, University of Guelph, Guelph, ON N1G 2W1 Canada; 2 L.H. Gray & Son Limited, Strathroy, ON Canada; 3 Department of Animal Sciences, Behavioural Ecology Group, Wageningen University, PO Box 338, 6700 AH Wageningen, The Netherlands

**Keywords:** Animal behaviour, Animal physiology

## Abstract

Maternal effects have been reported to alter offspring phenotype in laying hens. In this study, we investigated the effects of maternal environment and maternal age on egg traits and offspring development and behaviour. For this, we ran two experiments. First (E1), commercial hybrid hens were reared either in aviary or barren brooding cages, then housed in aviary, conventional cages or furnished (enriched) cages, thus forming different maternal housing treatments. Hens from each treatment were inseminated at three ages, and measures of egg composition, yolk testosterone concentration and offspring’s development, anxiety and fearfulness were assessed. In experiment 2 (E2), maternal age effects on offspring's growth and behaviour were further investigated using fertile eggs from commercial breeder flocks at three different ages. Results from E1 showed that Old hens laid heavier eggs with less yolk testosterone and produced offspring with fewer indicators of anxiety and fearfulness. Maternal rearing and housing affected egg traits, offspring weight and behaviour, but not in a consistent way. Effects of maternal age were not replicated in E2, possibly due to differences in management or higher tolerance to maternal effects in commercial breeders. Overall, our research confirms that maternal age and maternal environment affects egg composition, with maternal age specifically affecting yolk testosterone concentration, which may mediate physical and behavioural effects in offspring.

## Introduction

The phenotype of the laying hen is primarily determined by the combination of her genotype, environment and early-life history. In addition, environmental stressors experienced by the mother can affect her offspring. In natural populations of animals, the Predictive Adaptive Hypothesis suggests that early experience, including cues from the mother, result in an adult phenotype that is better adapted to environmental conditions when conditions experienced by mother and offspring or those experienced early and later in life are similar (environmental matching)^1–3^. In the egg industry, the term “environment” comprises a series of aspects that include the type of rearing and adult housing systems (which range from confined and barren to enriched and highly complex), population density and husbandry that the birds are subjected to, from hatch to end of lay. In contrast to wild avian populations, domesticated hens have been selected for high rates of egg laying, predation pressure is non-existent and their nutrition is optimized for life stage and productivity. However, domesticated birds still experience a variety of stressors, and many of the mechanisms underlying developmental plasticity are still at play, although no longer relevant to offspring life history or reproductive fitness.

In egg production systems, all laying hens are descendants of layer breeder flocks. The breeder hen’s life experience, whether positive or negative, can affect the behaviour and stress susceptibility of laying hens through epigenetic effects^4–6^ or changes in the concentration of hormones, nutrients or other egg components deposited by the mother in her eggs^7,8^. Over the years, research in layer breeders has resulted in advances in management, nutrition and genetics; however, there is a paucity of literature regarding the mechanisms, causes and consequences of maternal effects for this group of birds. Given that the global population of commercial layers is 7.6 billion animals, and each layer breeder produces approximately 115 offspring^9^, maternal effects in layer breeders can potentially affect innumerous hens.

Studies on commercial hens have consistently linked housing environment with short- and long-term effects on physical and cognitive traits. For example, hens reared in aviary showed improved bone and muscle growth^10,11^, better use of space^12,13^ and reduced fearfulness^14^ and stress response^15^ in comparison to birds reared in conventional brooding cages. Similarly, adult housing was shown to affect behaviour and stress response, with hens reared in conventional cages and housed in furnished cages being less stressed than hens both reared and raised in conventional systems^16,17^. Recently, offspring effects related to maternal rearing and housing condition have also started to be reported. A multifactorial analysis of laying production, production of hatching eggs, and the number of waste eggs showed that layer breeders reared in aviary and housed on litter had better results compared to cage-reared birds^18^. Additionally, our research group reported that maternal environment affected offsprings’ social behaviour and stress response, with aviary-reared mothers having less emotional offspring, whereas aviary-housed mothers had chicks with a lower stress response^19^.

Changes in the concentration of egg hormones and nutrients have been suggested as mediators for maternal effects in oviparous species^7,8^. Research has shown that maternal experiences can lead to changes in egg composition that, in turn, might affect offspring phenotype^8^. Hens subjected to a moderate heat challenge for five consecutive weeks laid eggs with higher concentrations of yolk steroid hormones (progesterone, testosterone and estradiol) and had lighter and calmer offspring than the control group^20^. Similarly, in the Japanese quail, mothers subjected to stressful events, such as sudden movement or unpredictable noise, laid eggs with higher yolk testosterone and progesterone, and produced chicks more sensitive to social separation (i.e., vocalized more during emergence and open-field tests)^21^. Androgen hormones such as testosterone have been suggested to be the main mediator of maternal effects in poultry species^22^; however, other egg components have also been investigated (glucocorticoid hormones^23^, thyroid hormones^24,25^, antioxidants^26^ and immunoglobulins^27^).

Another potential source of maternal effects in laying hens is maternal age. Across many taxa, a reduction in quality, viability and lifespan of offspring is commonly observed as parental age increases. This phenomenon is referred to as the Lansing effect, and the underlying mechanisms are not well understood^28^. As a hen gets older, the size and quantity of solid content in her eggs naturally increase^29^. In addition, yolk testosterone concentration may decrease over time, such as seen in the Japanese quail^30,31^. Ageing has also been linked to changes in yolk fatty acid profile^32,33^ and late embryonic mortality, hatchability, and body weight in the offspring of broiler breeders^34^. Although the current study is the first of its kind to assess how ageing affects yolk testosterone concentration in laying hens, a previous publication from our research group reported that maternal age increased both the endocrine and behavioural response of the offspring to manual restraint and affected different aspects of injurious social behaviour in laying hens^19^. Layer breeder flocks typically start producing offspring at around 20 weeks of age and remain in production until around 70 weeks of age, reaching an optimized performance (based on rate of egg production, hatchability and chick weight) at approximately 44 weeks^35,36^. Therefore, layer breeder’s age may also be a source of maternal effects in the offspring.

The current study is part of a more comprehensive research project on maternal effects^19,37–39^ that aim at investigating how maternal age, maternal rearing experience and maternal adult housing system affect their offspring’s behaviour and stress response. For this, we conducted two experiments. In the first longitudinal study (E1), two cohorts of commercial hybrid (Lohmann Selected Leghorn-Lite) hens were reared and housed in five housing system combinations and were inseminated at three ages (25, 44 and 68 woa), producing six offspring flocks. Measurements of egg composition, yolk testosterone concentration, and offspring growth and behaviour were assessed. To test the replicability of any maternal age effects found in E1 in commercial breeder flocks (parent stock), we conducted a second cross-sectional experiment (E2) in which hatching eggs from layer breeders at three age groups (“Young” (25–27 woa), “Ideal” (42–46 woa) and “Old” (68–72 woa)) were obtained from various commercial flocks across Canada and the United States. Data collection included measures of body weight and behaviour. We hypothesized that maternal effects related to age and environment would be found in E1 and predicted the replicability of maternal age effects in E2. Specifically, we predicted that older hens would lay bigger eggs with less yolk testosterone and produce offspring with increased behavioural response^19,30,31^. We also predicted that maternal age would have stronger effects on offspring phenotype than maternal environment due to more significant and consistent changes in egg composition. Lastly, we predicted that hens raised and housed in similar environments (e.g., aviaries) would be better adapted in comparison with hens from different rearing and housing combinations (e.g., reared in aviary and housed in conventional cage) and that these differences would further affect the phenotype of offspring.

## Methods

This study was carried out in compliance with the ARRIVE guidelines and birds were treated in accordance with the Canadian Council on Animal Care. All procedures were approved by the University of Guelph Animal Care Committee (Animal Utilization Protocol #1947).

### Experiment 1 (E1)

#### Parent stock

The methodology of this experiment and details on rearing and housing environments have been further described in Peixoto et al.^19^. In short, two cohorts of Lohmann Selected Leghorn Lite (LSL-Lite) pullets, were obtained from a commercial hatchery at one day of age and transferred to the University of Guelph’s Arkell Poultry Research Station. Immediately upon arrival, 408 of the chicks were housed in conventional barren brooding cages (CC, 8 birds per cage, 290 cm^2^/bird) with wire floor, nipple drinkers and a feeder trough. The remaining birds were assigned to a tiered pullet rearing aviary (Av, 778 birds, 754 cm^2^/bird) enriched with perches, terraces and access to wood shavings as litter.

At 16 weeks of age, 614 pullets from Av rearing were randomly selected and transferred to conventional cages (CC; N = 6 cages of 8 hens), furnished cages (FC; N = 3 cages of 60 hens) and an adult aviary system (Av; N = 1 group of 370 hens) located in different rooms. A sample of 244 pullets from CC rearing was randomly selected and transferred to CC (N = 6 cages of 8 hens) or FC (N = 3 cages of 60 hens). Birds from the same rearing treatments were housed together as adults. These sample sizes were repeated in the second cohort.

The conventional cages for adult hens were wire structures with nipple drinkers and an external feed trough stocked at 503 cm^2^ per hen. The furnished cages were larger, provided more space per individual hen (750 cm^2^ per hen) as well as an enclosed nest, perches and a scratch mat. The adult aviary provided the most space (1344 cm^2^ floor space per hen) and had 2 elevated tiers on which feed troughs, nipple drinkers, perches and nest boxes were located. The hens also had access to a litter floor with wood shavings.

**Figure 1:**
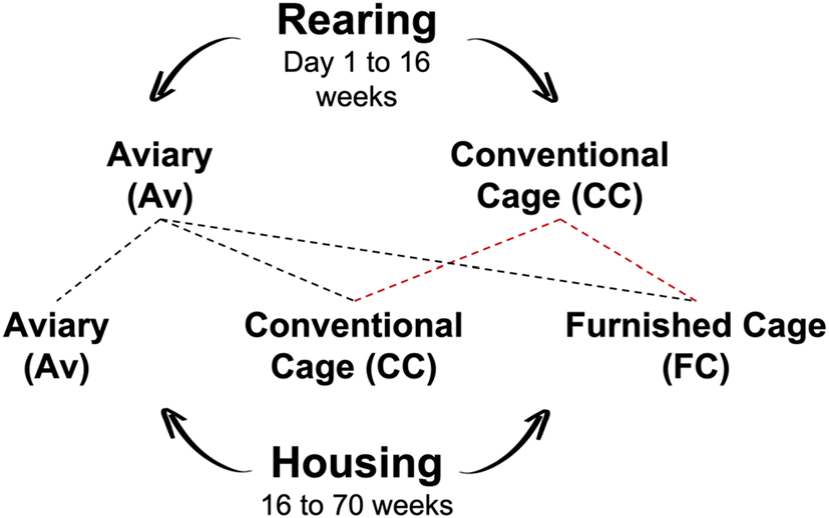
(From Peixoto et al.^19^). Experiment 1. The combination of maternal rearing and housing experiences formed five treatment groups: Trt1 (Av × Av), Trt2 (CC × CC), Trt3 (Av × CC), Trt4 (CC × FC), Trt5 (Av × FC). This was repeated for two cohorts of hens. Since pullets reared in cages have difficulty adapting to aviary housing, only pullets reared in aviaries were transferred to adult aviary housing.

Pullets reared in conventional rearing cages typically do not fare well when transferred to a complex aviary system^13^. Because of this, our experimental design was an incomplete factorial, with each rearing and housing combination considered a treatment (Fig. 1). A sample of 96 hens from each treatment were randomly selected and inseminated with pooled semen from a contemporary group of White Leghorn males: young (25 weeks), ideal (44 weeks) and old (68 weeks). Eggs from each maternal age, treatment and flock were collected and stored at 4 °C until incubation.

#### Egg composition and yolk testosterone concentration

A subset of eggs from each treatment and cohort were collected 2 days prior to each insemination and subsequently analyzed for egg quality traits (N = 1444) and yolk testosterone (N = 441). Eggs were weighed, broken, and the yolks were separated from the albumen. Before weighing, the chalazae were removed with a forceps and all yolks were rolled on a paper towel to remove adhering albumen. The shells were carefully washed and dried for 48 h in a drying oven at 21 °C and then weighed. Albumen weight was determined by subtracting yolk and shell weight from the original egg weight.

Testosterone concentration in yolk was analysed via enzyme immunoassay (EIA; antibody against testosterone-3-CMO), and followed an adapted methodology from Henriksen et al.^40^. Egg yolks were homogenized to ensure adequate mixing of hormones from different regions of the yolk sphere. Samples were extracted by mixing 0.15 g of yolk with 0.6 ml of double-distilled water and 3 ml of methanol were added. Solution was homogenized with a vortex placed in a walk-in cold room at 2 °C overnight. On the next day, solution was placed in − 20 °C freezer for 2 h to precipitate apolar lipids, that were transferred into a new vial. The remaining solution was centrifuged at 2500*g* for 15 min at 4 °C and stored in aliquots of 2 ml. The minimum detectable hormone level was 0.59 ng per g of yolk.

#### Offspring management and data collection

Offspring were incubated and hatched in commercial incubators at the University of Guelph’s Arkell Poultry Research Station. Chicks were sexed and individually wing-banded at hatch. Each cohort had four replicate groups of progeny (7 males and 7 females/each) per treatment and maternal age (N = 1680). Replicates were identically reared to 15 weeks in 20 floor pens (3.72 m^2^) that had a perch (length: 155 cm) and were bedded with litter. Offspring were weighed at hatch and 91 days of age. Behavioural tests were performed as described below. The test order for each test was balanced across treatment and time of day in order to minimize effects of circadian rhythm on results.

#### Social isolation test

The social separation of young chicks from their conspecifics produces an increase in distress vocalizations^41^, allowing for measurement of anxiety-related behaviours. Following the methodology proposed by Sufka et al.^42^, chicks at 5–6 and 32–33 days of age (N = 960) were individually placed into a sound-proof box (63.5 cm high × 63.5 cm deep × 63.5 cm wide) where their vocalizations were recorded (for pictures of the box and further details on methods, please see Peixoto et al.^38^). Each chick was tested at one age only. The test lasted 5 min and was conducted from 08:00 to 12:00 h and from 14:00 to 18:00 h in a quiet room close to their home pen. Vocalizations were recorded, saved as a MPEG-4 file using the Voice Memos application (Apple, Cupertino, USA). One observer blind to treatment, used WavePad (NCH Software, Greenwood Village, USA) to visualize and count the calls. Vocalizations were differentiated across four relative ranges of intensity, and those in the lowest range (< 18db) were excluded. All other calls were considered to be distress vocalizations and summed to calculate Total Distress Vocalizations (TDV).

#### Novel object test

The novel object test measures the conflicting motivation to approach or avoid a potentially dangerous stimulus and it has been traditionally used as an indicator of fear^43^. Following the methodology proposed by de Haas^44^ and based on the Welfare Quality assessment^45^, the novel object test was conducted at 3, 5 and 10 weeks of age. Each group of progeny (N = 60 pens with 14 birds/each) was exposed to a wooden box (5 × 5 × 2 cm) covered with coloured tape (green, yellow, red and white) in a striped pattern. The novel object was placed on the floor inside the birds’ home pen. A camcorder (Panasonic HC-V180K) was suspended from the ceiling, providing a complete overhead view of the pen. Instantaneous scan samples were taken every 10 s by one observer blind to treatment. The number of birds in proximity to the object were recorded at each time point. Latencies to approach and peck, respectively, were defined as the time points at which at least three birds were in close proximity of the object (one bird length: < 25 cm and two bird length: < 50 cm) and were observed to touch/peck the object.

#### Tonic immobility test

The Tonic Immobility (TI) methodology initially proposed by Jones^46^ and previously described by Peixoto et al.^37^ was used to measure fearfulness. At 9 weeks of age, two males and two females from each replicate group (N = 480) were individually caught, moved into a quiet nearby pen and placed on their back in a V-shaped cradle, where one of two experimenters gently applied pressure on their sternum. If birds became immobile for a minimum of 10 s, it was considered a successful induction. If not, up to five consecutive attempts to induce TI were performed. Each test lasted 10 min, or until the bird stood up. Testing was recorded using a camcorder (Panasonic HC-V180K) positioned perpendicularly to the cradle. The camera was plugged to a monitor located out of the birds’ sight in order to reduce effect of experimenter on the bird. Behaviours were analyzed from videos by one trained observer blind to treatment. Data collection included latency to look around, latency to stand up, number of vocalizations emitted during the test and number of attempts needed to attain a successful induction.

### Experiment 2 (E2)

#### Parent stock, offspring management and data collection

In our second experiment, we aimed to further investigate the effects of maternal age in layer breeders obtained from commercial settings. For this, fertile eggs were acquired from 9 unique, commercial LSL-Lite White Leghorn layer breeder flocks located in Ontario, Quebec and New Brunswick, Canada and from Pennsylvania, USA. Breeder flocks were loose-reared and housed in floor barns. At the time of egg collection, 3 of the flocks were Young (25–27 woa), 3 were Ideal (42–46 woa) and 3 were Old (68–72 woa). All of the fertile eggs were shipped to University of Guelph’s Arkell Poultry Research Station within the same week where egg storage, incubation and hatch occurred together, following the procedures previously described. From each parent flock, 2 replicates of progeny (7 males and 7 females; n = 28 per flock; 3 flocks/age; N = 252) were identically reared to 42 days of age.

Offspring were subjected to identical husbandry and housing as E1. Data collection included body weight at hatch and 41 days of age (N = 252) and half of the chicks were subjected to a social isolation test at 5–6 days of age (N = 126) which followed the methodology previously described.

### Data analyses

The Glimmix procedure of SAS 9.4 (SAS Institute, Cary, NC) was used to perform all statistical analyses. For E1, a mixed model analysis of variance was used to the fixed effects of sex (when applicable), maternal treatment (T1–T5: the combination of maternal rearing × adult housing), maternal age, maternal treatment × maternal age, maternal treatment × sex and maternal age × sex. Random effects included cohort, home-pen nested within room and person applying the test when applicable. When appropriate, statistical models accounted for repeated measures using the most applicable covariance structure in Glimmix. LSMeans with the Tukey’s adjustment were used for post hoc multiple comparisons of fixed effects. Because of the incomplete factorial design, maternal rearing and housing could not be included in the model as fixed effects. Therefore, post-hoc contrast comparisons were used to test the effects of maternal rearing (Av or CC) and housing (Av, CC or CF). Data from the social isolation and tonic immobility tests were lognormally transformed to meet the assumption of a normal distribution of residuals. In addition, statistical analyses of social isolation test and body weight included chick age as a fixed effect.

In E2, we used a generalized mixed model analysis of variance to test the effects of sex and maternal age. Random effects included flock of origin and home-pen of the tested bird nested within room. Tests for normality included Shapiro–Wilk and Anderson Darling measurements in conjunction with visual plots for all analyses and statistical significance was defined as P < 0.05 for both studies. Unless otherwise indicated, LSMeans and standard errors are given in the tables and figures.

## Results

### Egg composition and yolk testosterone concentration

Egg composition and yolk testosterone concentration for E1 are presented in Table 1. Results were as expected, with an increase (P < 0.0001) in egg weight (g) and yolk (%) as the hens aged. Hens both reared and housed in aviary systems (Trt 1, AV × AV) produced eggs of different interior composition with less (P < 0.0001) yolk (%), and more (P < 0.0001) albumin (%) content than all treatments. A statistical contrast analysis determined that hens who were reared in aviaries produced heavier (P ≤ 0.0442) eggs than hens that were conventionally reared.

**Table 1:** Egg composition and yolk testosterone concentration.

	Experiment 1							
Variable					Overall *P*-value			
	EW	YW	AW	YT	EW	YW	AW	YT
	Egg weight (g)	Yolk weight (%)	Albumin weight (%)	Yolk testosterone (ng/g)				
**Maternal age**					< 0.0001	< 0.0001	< 0.0001	0.0260
Young	56.9 ± 0.20^c^	24.7 ± 0.12^b^	63.5 ± 0.15^a^	1.46 ± 0.14^a^				
Ideal	61.5 ± 0.28^b^	28.5 ± 0.15^a^	59.9 ± 0.14^c^	1.31 ± 0.03^ab^				
Old	64.2 ± 0.34^a^	28.4 ± 0.16^a^	60.35 ± 0.16^b^	1.06 ± 0.07^b^				
**Maternal treatment**					< 0.0001	< 0.0001	< 0.0001	0.7605
Trt 1 (AV × AV)	61.33 ± 0.61^a^	26.44 ± 0.30^b^	62.13 ± 0.29^a^	1.39 ± 0.07				
Trt 2 (CC × CC)	60.55 ± 0.88^b^	27.65 ± 0.47^a^	60.62 ± 0.40^b^	0.21 ± 0.17				
Trt 3 (AV × CC)	60.92 ± 0.87^ab^	27.55 ± 0.48^a^	60.85 ± 0.41^b^	1.25 ± 0.19				
Trt 4 (CC × FC)	60.31 ± 0.73^b^	27.66 ± 0.45^a^	60.94 ± 0.42^b^	1.34 ± 0.13				
Trt 5 (AV × FC)	60.66 ± 0.71^ab^	27.55 ± 0.47^a^	60.86 ± 0.44^b^	1.20 ± 0.08				
**Materal age** × **maternal Trt**					0.4483	0.4483	0.5200	0.9677

LSMean ± SEM for egg quality traits, egg composition and yolk testosterone of eggs from hens in Experiment 1 collected at three different ages.

^abc^Values within a column for Maternal Age and Maternal Rearing × Adult Housing treatments with different superscripts differ P < 0.05.

A significant effect of maternal age on yolk testosterone content showed that Young hens deposited significantly (P = 0.0260) more yolk testosterone (ng/g yolk) than Old hens. There was no significant effect of either maternal rearing or housing environment on yolk testosterone content. Additionally, there were no interactions between maternal age, or the maternal rearing × adult housing environment.

### Offspring growth

Growth and body weight data for progeny from both E1 and E2 are presented in Table 2. In E1, offspring hatch weight increased (P < 0.0001) with maternal age; however, the inverse was found for average daily gain (ADG; see below) and final bodyweight. When hens were Old, their progeny had slower ADG (P < 0.0032) and lower final bodyweight (P = 0.0094) at 91 days of age, than the progeny that they produced when they were at Young or Ideal breeder ages. Maternal rearing and adult housing both affected hatch weight; with Trt 2 (CC × CC) progeny having lower (P ≤ 0.0076) hatch weights than all other treatments. A statistical contrast analysis determined that there was a significant effect of maternal rearing on hatch weight in E1, with heavier (P = 0.0409) hatch weights from Aviary-reared hens.

$$ADG=\frac{{BW}_{91}-{BW}_{1}}{90\;{\text{days}}}$$

ADG is the average daily gain; ${BW}_{91}$ is the body weight at 91 days of age; ${BW}_{1}$ is the body weight at 1 day of age.

**Table 2:** Offspring growth.

Variable				Overall *P*-value		
	HW	BW	ADG	HW	BW	ADG
	Hatch weight (g)	Weight 91 days (g)	Avg daily gain (g)			
**Experiment 1**						
Maternal age				< 0.0001	0.0094	0.0032
Young	38.4 ± 0.09^c^	1283.4 ± 25.4^a^	13.7 ± 0.28^a^			
Ideal	39.9 ± 0.13^b^	1275.6 ± 24.5^ab^	13.6 ± 0.27^ab^			
Old	41.2 ± 0.18^a^	1257.9 ± 24.7^b^	13.4 ± 0.28^b^			
Maternal treatment				0.0076	0.3970	0.4429
Trt 1 (AV × AV)	40.0 ± 0.26^a^	1284.5 ± 32.5	13.7 ± 0.36			
Trt 2 (CC × CC)	39.2 ± 0.21^b^	1260.0 ± 32.6	13.4 ± 0.36			
Trt 3 (AV × CC)	40.1 ± 0.26^a^	1274.4 ± 30.6	13.6 ± 0.36			
Trt 4 (CC × FC)	39.8 ± 0.22^a^	1272.8 ± 32.3	13.5 ± 0.35			
Trt 5 (AV × FC)	40.1 ± 0.27^a^	1269.6 ± 33.2	13.5 ± 0.36			
Sex				0.0046	< 0.0001	< 0.0001
Cockerel	40.1 ± 0.16^a^	1486.9 ± 5.3^a^	15.9 ± 0.06^a^			
Pullet	39.6 ± 0.15^b^	1057.7 ± 4.6^b^	11.2 ± 0.05^b^			
Maternal age × sex				0.8545	0.4878	0.4681
Materal age × Trt				0.0791	0.6628	0.6732
Trt × Sex				0.8059	0.4808	0.4833
**Experiment 2**						
Maternal age				0.3103	0.0068	0.0057
Young	38.6 ± 0.39	440.2 ± 11.7^c^	9.8 ± 0.28^b^			
Ideal	40.4 ± 0.55	466.3 ± 14.6^a^	10.4 ± 0.36^a^			
Old	39.8 ± 0.58	456.3 ± 14.3^b^	10.2 ± 0.35^ab^			
Sex				0.6045	< 0.0001	< 0.0001
Cockerel	39.7 ± 0.44	483.9 ± 5.5^a^	10.8 ± 0.13^a^			
Pullet	39.5 ± 0.52	424.6 ± 3.1^b^	9.4 ± 0.07^b^			
Maternal Age × Sex				0.2020	0.1211	0.1120

Means ± SEM for hatch weight, body weight, and average daily gain for progeny from Experiments 1 and 2.

^abc^Values within a column for Maternal Age and Maternal Rearing × Adult Housing treatments with different superscripts differ P < 0.05.

The maternal age effect on growth patterns for progeny from E2 study did not match those of progeny from E1. In the second experiment, hatch weight was not affected by maternal age, and progeny from young breeder hens had the lowest ADG (P = 0.0068) and final body weight at 41 days of age (P = 0.0057) compared to those of older breeder hens.

### Social isolation test

**Figure 2:**
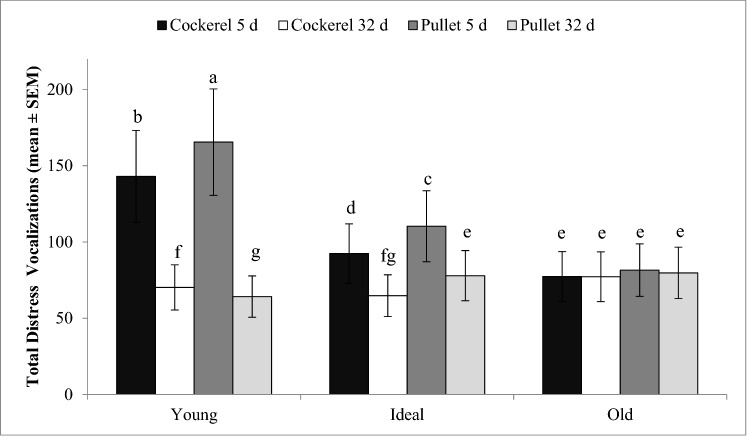
LSMeans ± SEM total distress vocalizations (TDV) of 5 day old and 32 day old cockerels and pullets during the 5-min social isolation test. There was an interaction between maternal age × chick age × sex (P < 0.001). Behavioral differences are indicated by different superscripts (P ≤ 0.002)^a–g^.

For E1, when chicks were tested at 5–6 days of age, maternal age had a significant effect on TDV (P < 0.0001); progeny of Young breeder hens had more TDV than those from Ideal, and Old. Chicks tested at 5–6 days of age vocalized more during the test than chicks tested at 32–33 days of age (P < 0.0001). There was also an interaction between maternal age × chick age × sex (P < 0.001). When their mothers were Young and Ideal ages, pullets vocalized more during the test than cockerals, but TDV did not differ between sexes when their mothers were old (Fig. 2). It was determined that this test is best conducted when chicks are younger and vocalize more in comparison to older ages.

**Figure 3:**
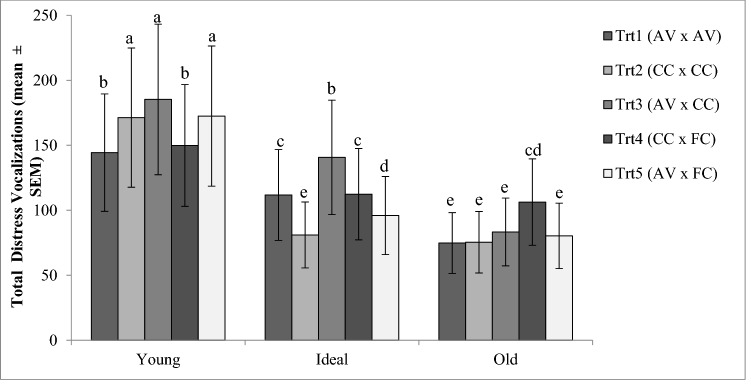
Social isolation test. LSMean ± SEM values for total distress vocalizations during 5-min sessions, for Experiment 1 progeny from the different Maternal Rearing × Adult Housing treatments, and from three maternal ages tested at 5 days of age. Behavioral differences are indicated by different superscripts (P < 0.001^a–e^).

Mean TDV for E1 progeny from hens in the different maternal treatments at the different maternal ages are illustrated in Fig. 3. There was a significant interaction between Maternal Age by Treatment (P < 0.0001). Trt 3 (AV × CC) had the highest TDV count at Young and Ideal maternal age. The statistical contrast analysis determined that there was an overall effect (P = 0.0068) of maternal rearing on TDV whereby progeny from Conventional reared hens had a lower TDV count than progeny of Aviary-reared hens. There was also an effect of Adult housing, where progeny from hens housed in both Conventional and Furnished housing vocalized more (P < 0.0001) than progeny from mothers housed in Aviary.

For E2, a similar trend for maternal age on TDV was observed (P = 0.076), although values from chicks in this population were much lower than those from E1. TDV during the 5-min test were 74.7, 60.8, and 41.7 (pooled SEM =  ± 8.7) for progeny of Young, Ideal and Old breeder hens, respectively.

### Novel object test

Latency to approach the novel object decreased (P ≤ 0.0395) with maternal age of the breeder hens (Table 3). There was no effect of Maternal Rearing × Adult Housing treatment; nor were there any interaction effects with either maternal age or progeny age. Statistical contrasts did not identify any overall effects of either maternal rearing or adult housing. However, offspring age resulted in significant (P < 0.0001) differences for all variables measured. At three weeks of age, offspring failed to peck the novel object and had the longest time to approach the novel object. Behavioural response to the novel object decreased with offspring age (P < 0.0001).

**Table 3:** Novel object test.

	Experiment 1					
Variable				Overall *P*-value		
	P-NO	O-NO	T-NO	P-NO	O-NO	T-NO
	Latency to peck the NO	Latency to one bird length from the NO	Latency to two bird lengths from the NO			
**Maternal age**				0.6810	0.0097	0.0395
Young	94.0 ± 3.4	49.8 ± 4.2^a^	18.9 ± 2.4^a^			
Ideal	95.4 ± 3.3	41.9 ± 3.7^ab^	15.0 ± 2.0^ab^			
Old	91.3 ± 3.4	38.9 ± 3.6^b^	15.0 ± 2.4^b^			
**Maternal treatment**				0.8391	0.9624	0.9961
Trt 1 (AV × AV)	94.0 ± 4.1	45.0 ± 4.4	18.0 ± 3.4			
Trt 2 (CC × CC)	95.6 ± 4.4	43.1 ± 4.8	17.0 ± 3.3			
Trt 3 (AV × CC)	91.9 ± 4.6	41.2 ± 4.6	14.5 ± 2.2			
Trt 4 (CC × FC)	95.6 ± 4.2	43.0 ± 4.9	14.2 ± 2.6			
Trt 5 (AV × FC)	90.8 ± 4.5	45.6 ± 5.3	17.8 ± 3.1			
**Offspring age**				< 0.0001	< 0.0001	< 0.0001
3 weeks	120.0 ± 0.0^a^	94.3 ± 3.0^a^	38.4 ± 3.0^a^			
5 weeks	100.8 ± 2.8^b^	23.0 ± 1.2^b^	9.1 ± 0.5^b^			
10 weeks	60.0 ± 3.2^c^	13.3 ± 1.0^c^	1.5 ± 0.1^c^			
Materal age × Trt				0.1715	0.1719	0.0505
Maternal age × offspring age				0.5486	0.1833	0.8924
Trt × offspring age				0.9842	0.6693	0.1507

Means ± SEM for latency to peck and latency to approach a novel object for progeny from Experiment 1.

^abc^Values within a column for Maternal Age and Progeny Age with different superscripts differ P < 0.05.

### Tonic immobility

Means ± SEM for variables measured during TI for E1 progeny are presented in Table 4. Maternal age affected the number of vocalizations during the test, with progeny from Ideal breeder hens vocalizing more (P = 0.0034) than progeny from either Young or Old breeder hens. Maternal rearing × adult housing did not affect any of the analyzed measures, but birds from Trt 2 (CC × CC) and Trt 4 (CC × FC) showed a tendency to take longer to look around (P = 0.0659). Overall, pullets were less fearful than males; they needed more attempts to induce TI (P = 0.0003), vocalized more and showed shorter latency to look around during the test.

**Table 4:** Means ± SEM for number of attempts to induce, number of vocalizations, latency to look, and latency to right during a tonic immobility test conducted on progeny from Experiment 1 at 9 weeks of age.

	Experiment 1							
Variable					Overall *P*-value			
	ATI	V	LL	LR	ATI	V	LL	LR
	# Attempts to induce	# Vocalizations	Latency to look	Latency to right				
**Maternal age**					0.0910	0.0034	0.3242	0.8098
Young	1.77 ± 0.11	2.76 ± 0.5b^ab^	75.7 ± 7.47	174.4 ± 11.78				
Ideal	2.03 ± 0.12	3.01 ± 0.78^a^	85.4 ± 9.46	189.0 ± 14.33				
Old	1.66 ± 0.10	2.69 ± 0.67^b^	63.3 ± 6.01	172.1 ± 11.4				
**Maternal rearing** × **adult housing**					0.7775	0.0980	0.0659	0.2490
Trt 1 (AV × AV)	1.80 ± 0.14	2.91 ± 0.77	65.8 ± 7.86	182.8 ± 16.50				
Trt 2 (CC × CC)	1.84 ± 0.14	3.57 ± 1.15	85.2 ± 11.65	182.8 ± 16.72				
Trt 3 (AV × CC)	1.70 ± 0.12	3.26 ± 1.02	63.5 ± 8.41	188.1 ± 17.21				
Trt 4 (CC × FC)	1.85 ± 0.14	1.78 ± 0.60	97.8 ± 12.3	187.9 ± 15.71				
Trt 5 (AV × FC)	1.91 ± 0.15	2.52 ± 0.69	60.44 ± 8.25	149.0 ± 14.05				
**Sex**					0.0003	< 0.0001	< 0.0001	0.7017
Cockerel	1.59 ± 0.07^b^	0.17 ± 0.05^b^	92.2 ± 7.22^a^	178.7 ± 10.2				
Pullet	2.05 ± 0.10^a^	5.74 ± 0.77^a^	54.9 ± 4.52^b^	177.8 ± 10.1				
Materal age × sex					0.1647	< 0.0141	0.3414	0.5522
Maternal age × Trt					0.4415	< 0.0001	0.7681	0.1832
Trt × Sex					0.4981	< 0.0001	0.1637	0.0171

^ab^Values within a column for Maternal Age and Sex with different superscripts differ *P* < 0.05.

Statistical contrasts identified an overall effect of maternal rearing whereby progeny from breeder hens reared in aviaries, as compared to conventional cages, had a shorter (P = 0.0045) latency to look around during TI.

## Discussion

In the first experiment (E1) of the current study, we observed that aviary-reared hens laid heavier eggs and had offspring that were heavier at hatch, more anxious in the social isolation test but had a shorter latency to look around when in tonic immobility. Housing experience during egg production also affected offspring behaviour, with mothers housed in an aviary producing less anxious offspring. Still in E1, we found that Old hens laid heavier eggs with more yolk percentage, less yolk testosterone and hatched heavier chicks in comparison with Young hens. Behaviourally, the offspring of Old hens were less anxious and less fearful.

The importance of rearing environment on physical, behavioural and cognitive development of laying hens has been vastly reported^13,47^. In agreement with previous studies^48–51^, our research shows that maternal rearing environment affected offspring behaviour more so than the current housing conditions of the mother. In E1, aviary-reared hens had chicks that vocalized more during social isolation and moved quicker out of tonic immobility, indicating higher levels of anxiety but lower fearfulness. As previously reported^19^, the offspring of aviary-reared hens from this same population also showed less displacement preening (an indicator of stress) during a social stress test in comparison with the offspring of cage-reared hens. In addition, E1 showed that aviary-reared hens laid heavier eggs and, consequently, hatched heavier offspring. A recent study of aviary rearing of layer breeder flocks found a sex-dependent effect on offspring hatching weight^18^. Cockerels hatched from breeders reared in aviaries were heavier than those whose mothers were reared in cages—a maternal effect not observed for pullet chicks. However, contrasting results from the literature suggest that other factors, such as genotype and diet, are more likely to affect egg weight than maternal rearing environment^52^. Although many of the mechanisms and pathways behind the effects of maternal rearing are still largely unknown, research has shown that rearing environment can change the DNA methylation profile of regulatory regions linked with gene expression in adult laying hens^49^. Therefore, similarly to other species^53^, epigenetic modifications may be the source of maternal effects developed during early life.

The combination between rearing and housing environments increased yolk percentage in Trt 1 (AV × AV) and impaired offspring hatch weight in Trt 2 (CC × CC). Furthermore, contrast analyses showed that offspring from aviary-housed mothers (during egg production) vocalized less during social isolation, indicating less anxiety than the offspring from cage-reared mothers. In agreement with these findings, we previously reported that offspring from mothers housed in aviary systems had lower stress responses (measured as plasma corticosterone concentration) in response to physical restraint^19^. Interestingly, maternal aviary housing caused opposite response of the offspring to social isolation than maternal aviary rearing. This discrepancy confirms the importance of time of stress exposure and suggests that maternal effects are caused by different mechanisms over time. While stressors experienced by the pullet during development might affect her offspring primarily through epigenetics^54^, stress exposure during adulthood seems to lead to changes in concentration of egg yolk hormones, as seen in lizards^55^, quails^56,57^ and laying hens^20,58^. Traditionally, maternally transferred androgens such as testosterone have been suggested as the primary mediator of these effects^22^, but research has also focused on other hormones including glucocorticoids^23^ and thyroid hormones^7^.

Although our analyses of egg composition failed to show an effect of maternal environment on egg yolk testosterone, one of our most significant findings is that the concentration of yolk testosterone progressively decreased as mothers aged, as seen in quails^30,31^. It has been suggested that the natural loss of reproductive function in ageing hens dampens testosterone production by cell layers that surround the growing oocyte^8^. In addition, ageing can influence the embryonic absorption and metabolism of yolk lipids, causing short- and long-term effects on offspring^32^. In E1, both egg weight and offspring hatch weight increased with maternal age, but the offspring of Old mothers displayed the lowest growth rates to 91 days of age and, consequently, the lowest body weight at that same age. Maternal age also affected offspring development in E2, with Ideal mothers producing heavier offspring than Old and Young at 41 days of age. Previous studies have linked the effects of pre-natal testosterone with offspring growth in chickens^59^ and passerine species^60^. Moreover, research on broiler breeders^61,62^ and turkeys^63^ further confirms the effects of maternal age on offspring development.

Regarding the effects of maternal age on offspring behaviour, Old mothers from E1 had chicks that vocalized less during social isolation and showed a shorter latency to approach a novel object, indicating less anxiety and fearfulness in comparison with offspring from Young mothers. Old mothers also showed a tendency to produce less anxious offspring in E2. We previously reported that maternal age affected stress response to physical restraint and injurious social behaviour that was possibly linked to the establishment of a social hierarchy in layers^19^. Although these effects are not yet fully understood, in ovo injections of testosterone were found to affect offspring plasma testosterone concentration and androgen receptor densities within the offspring brain^64^, suggesting a potential pathway for short- and long-term maternal effects.

Overall, most of the analyzed traits in E1 were not replicated in E2. This might be explained by the genetic differences between laying hens and layer breeders. While we used commercial hens as a model for layer breeders in E1, we directly tested layer breeders in E2. Therefore, results suggest that layer breeders are more resilient to maternal effects than laying hens, as previously reported^19,37,39^. Additionally, our study was performed during an Avian Influenza outbreak across North America that severely limited access to eggs from commercial breeder flocks, thus limiting E2’s sample size. As well, commercial breeder flocks are usually fed diets with different nutrient levels over the course of their laying period^36^ (phase feeding) which is a confound with maternal age and was not done in our experimental flock in E1. As mentioned previously, maternal nutrition has a significant impact on egg and chick characteristics. Additionally, the housing and social environments of breeder flocks (usually comprising thousands of birds and allowing a natural mating system of hens and roosters^36^) can also affect the endocrine status of the hen^65^, thereby affecting the hormone content of the layer breeder’s eggs^66^, and potentially masking maternal age effects. Finally, E1 was designed as a longitudinal study in a controlled research environment, whereas E2 was a cross-sectional field study with more differences in the life histories of the parent population that could add variation to the data^28^. It should be noted that paternal ages were the same for the mothers’ in both studies, and rates of egg laying and egg traits, which are the main forms of parental investment in domestic poultry, have been artificially selected for and are relatively uniform across flocks.

Regardless of maternal environment and maternal age, a sex effect was found in E1 during the social isolation and tonic immobility tests. Sexually dimorphic behaviour is primarily related to the effects of gonadal hormones on the nervous system^67^. Pullets vocalized more than cockerels during the social isolation test at 5 days of age, but this difference depended on age of their mothers. Interestingly, there was no difference between sex when their mothers were Old, when T levels and TDV were both lowest. In the TI test, pullets needed more attempts to be induced, took less time to look around, and vocalized more than males. These results suggest that female offspring are more anxious but less fearful than males, corroborating with previous studies^37,68^ (but see^38^).

Herein, we report that maternal age and maternal environment can affect offspring's development and behaviour when commercial hens are used as a model for breeding chickens. Maternal rearing and housing environment were found to affect anxiety and fearfulness, but in different ways and possibly through different mechanisms. Maternal ageing was shown to decrease the availability of yolk testosterone to the embryo, impair body weight at 91 days of age and decrease anxiety and fearfulness in offspring. A trend to similar effects on offspring behaviour (anxiety levels) were found in layer breeders. Overall, layer breeders seem to be more resilient to the effects of maternal age, but further studies are highly encouraged. In conclusion, we recommend that researchers and producers start noticing and reporting the age and housing condition of their birds’ parent stock and encourage farmers to note their flock’s parents' age to identify possible behaviour patterns and eventually anticipate productive issues.

## Data Availability

The data that support the findings of this study are available from the corresponding author upon reasonable request.
